# Seaweed’s Role in Energetic Transition—From Environmental Pollution Challenges to Enhanced Electrochemical Devices

**DOI:** 10.3390/biology11030458

**Published:** 2022-03-17

**Authors:** Susete Pinteus, Patrícia Susano, Celso Alves, Joana Silva, Alice Martins, Rui Pedrosa

**Affiliations:** 1 MARE—Marine and Environmental Sciences Centre, Polytechnic of Leiria, 2520-630 Peniche, Portugal; patricia_susano94@hotmail.com (P.S.); celso.alves@ipleiria.pt (C.A.); joana.m.silva@ipleiria.pt (J.S.); alice.martins@ipleiria.pt (A.M.); 2 MARE—Marine and Environmental Sciences Centre, ESTM, Polytechnic of Leiria, 2520-614 Peniche, Portugal

**Keywords:** climate change, critical raw materials, bioremediation, marine macroalgae, electronic waste, biochar, energy-storage devices, renewable energies, supercapacitors, rare earth elements

## Abstract

**Simple Summary:**

Earth is currently facing the effects of climate change in all environmental ecosystems; this, together with pollution, is the cause of species extinction and biodiversity loss. Thus, it is vital to take actions to mitigate and decrease the release of greenhouse gases to the atmosphere. The emergence of energetic transition from fossil fuels to greener energies is clearly defined in the United Nations 2030 agenda. Although this transition endorses the ambitious goal to supply greener energy for all developed societies, the increased demand for the minerals essential to develop cleaner energetic technologies has highlighted several economic and environmental issues. Currently, these minerals are mainly obtained by mining activities that generate high levels of soil and water pollution, coupled with the intensive use of water and hazardous gas release. On the other hand, the exponential increase of electronic waste derived from end-of-life electronic equipment is already raising environmental concerns due to heavy metal contamination as a result of their disposal. Thus, it is vital to develop sustainable and efficient strategies to mitigate energetic transition environmental footprints. This review highlights the use of seaweed biomass for toxic mineral bioremediation, recycling, and as an alternative material for greener energy-storage device development.

**Abstract:**

Resulting from the growing human population and the long dependency on fossil-based energies, the planet is facing a critical rise in global temperature, which is affecting all ecosystem networks. With a growing consciousness this issue, the EU has defined several strategies towards environment sustainability, where biodiversity restoration and preservation, pollution reduction, circular economy, and energetic transition are paramount issues. To achieve the ambitious goal of becoming climate-neutral by 2050, it is vital to mitigate the environmental footprint of the energetic transition, namely heavy metal pollution resulting from mining and processing of raw materials and from electronic waste disposal. Additionally, it is vital to find alternative materials to enhance the efficiency of energy storage devices. This review addresses the environmental challenges associated with energetic transition, with particular emphasis on the emergence of new alternative materials for the development of cleaner energy technologies and on the environmental impacts of mitigation strategies. We compile the most recent advances on natural sources, particularly seaweed, with regard to their use in metal recycling, bioremediation, and as valuable biomass to produce biochar for electrochemical applications.

## 1. Introduction

The planet Earth has been in constant change, forcing all living creatures to adapt and evolve. However, since the last century, it suffered by fast and aggressive changes due to human activity. With the worlds’ population increasing, and consequently increased demand for fossil-based energies for energy supply, a global phenomenon has arisen: global warming.

Scientific models predict that with the continued use of fossil-based energies, such as oil, coal and natural gas, the greenhouse gas concentrations will continue to rise, enhancing Earth’s temperature [1] and imposing dramatic irreversible changes in all ecosystems, leading to species extinction and thus threatening all humankind. As a result, it is imperative to change behaviors towards more sustainable and greener energy technologies. At the 2015 United Nations Climate Change Conference (UNCCC), representatives from 196 countries met in Le Bourget, France, and decided to limit the use of fossil-based energies, limiting the release of greenhouse gas emissions to the atmosphere [2]. More recently, within the European Green Deal, the European Union defined the ambition to be climate-neutral by 2050, establishing an economy with net-zero greenhouse gas emissions [3]. Thus, long-term low greenhouse gas emission strategies must be developed.

Global energy production and distribution has witnessed a progressive transformation from high emission fossil-based sources to variable renewable energy sources. By the end of 2020, the global share of electricity generation from renewables (solar, hydropower, and wind) reached about 29% [1]. According to the International Renewable Energy Agency (IRENA), to meet the objectives of the Paris Agreement on decarbonization of the power generation sector, at least 85% of the total power generation must be from renewables in 2050 [4]. With this urgent but ambitious goal, it is imperative to join efforts within the scientific community and industrial sectors to overcome the main challenges of energetic transition, particularly the sustainability and environmental footprint issues. Within this framework, this review aims to highlight the potential of seaweed as a vehicle to mitigate some of the negative impacts associated with energetic transition, namely for the bioremediation of heavy metal pollution, valuable metal recovery and recycling, and as carbon precursors for enhanced energy-storage devices.

## 2. Critical Raw Materials Demand

Decarbonization is a complex process that affects the entire structure of the world’s economy. To achieve this ambitious objective, solar and wind energies must reach an unprecedent scale, thus increasing the demand for raw materials, and in particular, the minerals needed to build wind turbines, solar panels and energy-storage devices [5,6]. Apart from the rising prices, currently there are no limitations on some minerals, such as copper, cadmium, selenium and nickel; however, other metals that are currently considered critical raw materials (CRMs) may be limited, thus limiting the development of clean energy technologies and slowing down the energetic transition progress [7].

High-tech products such as smartphones, laptops, electric vehicles, health apparatuses, solar panels, etc., require up to 50 different metals, including CRMs, which are currently irreplaceable (Table 1). These are deemed critical due to their economic importance, high-supply risk in several countries and current lack of substitutes.

To achieve the ambitious goal to be climate-neutral in 2050, the European Union has estimated that up to 60 times more lithium, 15 times more cobalt and 10 times more rare earth elements will be needed by 2050 to produce electric vehicle batteries and energy-storage devices, digital technologies and wind generators [6,9].

CRMs are therefore key metals for technological progress and for the functioning of the worlds’ economy; thus, different strategies must be put in action to increase the economic and environmental sustainability of these metals’ exploration and use. The exponential demand for these materials has a significant environmental impact, resulting from mining exploration and processing (coupled with high amounts of toxic waste produced and water consumption), putting serious pressure on environmental health. These activities are leading to the destruction of habitats and fertile land and resource depletion (particularly water) and are directly affecting human health. As a result, it is imperative to develop strategies to mitigate these actions’ side-effects by fine-tuning technology, recycling and decreasing the need for these materials by finding sustainable alternatives.

Recycling CRMs is a possibility with a real economic potential. However, despite different governmental initiatives to encourage recycling within a circular economy approach, CRM recycling is still extremely low, and it is inexistent for some metals, such as beryllium, borate, gallium, indium, niobium, phosphorus, scandium and silicon-metal [10]. As a result, it is extremely important to develop strategies for CRM recovery and recycling.

### Seaweed-Based Strategies for the Recovery of Critical Raw Materials

Critical raw materials, particularly rare earth elements (REEs), are critical strategic elements in the energetic transition framework. These are 17 elements comprising 15 lanthanides (Lanthanum (La), Cerium (Ce), Praseodymium (Pr), Neodymium (Nd), Promethium (Pm), Samarium (Sm), Europium (Eu), and Gadolinium (Gd)), plus Yttrium (Y) and Scandium (Sc). Due to their unique magnetic, electrochemical and luminescent properties, these are key components in a wide range of electronic devices, such as wind turbines, solar panels, electric vehicles, smartphones, computers, and medical equipment [11]. Although these minerals are found in the Earth’s crust, they are particularly concentrated in three mines in China, which makes China the dominant producer of REE, accounting for 97% of global production [12]. In addition, extraction of these metals involves the extraction of non-target toxics, such as fluorine and radionuclides such as ^238^U and ^232^Th, which significantly impacts worker health and ends up as waste, leading to soil and water contamination if not properly managed [13]. As the demand for these materials is exponentially rising, it is urgent to take actions to find more sustainable alternatives to obtain these valuable materials and to reduce equipment disposal impacts through recycling and pollution remediation; these objectives are clearly defined in the European Union goals for 2030 and 2050 [14,15].

Recently, new biotechnological approaches based on seaweed have been proposed to recover CRMs, including REEs, from contaminated waste (Table 2).

Due to their high value for new technology development and to their high toxicity for humans and the environment, several strategies have been developed to recover REEs from different polluted scenarios. These include costly techniques such as reverse osmosis, ion-exchange, solvent extraction, and electrochemical separation, which produce other toxic secondary products in the process that need to be addressed, thus increasing the processing costs substantially. On the other hand, none of these techniques have shown to be efficient in the uptake of REEs for industrial-scale purposes [16]. Compared with other pollutants, most of the REEs are present in low concentrations, significantly limiting their recovery from these complex mixtures [22].

Live seaweeds are known as efficient biosorbents of different pollutants, including polycyclic aromatic hydrocarbons (PAHs) [24], pesticides [25], antimicrobials [26] and heavy metals [27], among others [28]. These characteristics make seaweeds compelling organisms for bioremediation and CRM recycling. Recently, Pinto et al. (2020; 2021) observed that the green seaweed *Ulva lactuca* has a high capacity to absorb light rare earth elements (>60%), such as Y, La, Ce, Praseodymium (Pr), Nd, Eu, Gadolinium (Gd), Terbium (Tb) and Dysprosium (Dy); *Gracilaria gracilis* can absorb La, Ce, Pr, Gd and Nd; and *Fucus vesiculosus*, La, Ce and Eu [18,29]. Another study focusing in *Ulva* and *Gracilaria* species also showed that these seaweeds have a high ability to capture Nd from ecosystems [21]. In addition to these particular species, other seaweed species have also shown high ability to absorb REE (La, Ce, Pr, Nd, Eu, Gd, Tb, Dy and Y) from aqueous solutions, such as *Ulva intestinalis, Osmundea pinnatifida* and *Fucus spiralis* (with a bioconcentration factor of 2790, 1742 and 841, respectively) [17]. The red seaweed *Gracilaria gracilis* was able to remove 70% of 500 µg/L of several light rare earth elements (REE)—Y, Ce, Nd, Eu, and La—and further optimization allowed the recovery of 100% of all these elements [19].

Within a circular economy concept, the use of seaweed-derived compounds may offer additional advantages for scaling-up the process of remediation. Seaweeds are rich in bioactive ingredients that have a wide range of biotechnological applications; thus, using seaweed “waste” will enhance the economic viability of the process. Some examples of using seaweed biomass can be found in the literature. A work conducted by Keshtkar et al. (2019) showed that is possible to use dried seaweed biomass to recover La and Ce [16]. The authors increased the adsorption capacity of this seaweed biomass towards these ions by chemical modification through xanthation, and verified a maximum efficiency of 38.26 mg/g and 41.44 mg/g of La, and Ce, respectively. Seaweed’s carrageenans have also demonstrated potential to recover metallic compounds from the environment. A work conducted by Abdellatif et al. (2020) [30] developed magnetic aerogels with seaweed-based carrageenan and a polyamidoamine dendrimer, and verified a high ability to remove several heavy metallic ions from an aqueous source, particularly Co (99% recovery). By extracting polysaccharides from seaweeds for bioremediation purposes, it is possible to use the remaining biomass to extract bioactive compounds, thus maximizing the use of the same resource [31,32].

## 3. Electronic Devices’ End of Life—What Next?

Although extremely necessary, energetic transition brings serious environmental challenges. With the rapid growth of the electronic industry associated with green energies, there is an ever-growing challenge to develop strategies to mitigate the negative impacts of waste derived from end-of-life electric and electronic equipment (e-waste). It is estimated that in 2030 the global production of e-waste will be more than 70 million tons [33,34]. For instance, smartphones and computers contain numerous toxic elements, including hazardous hexavalent chromium (Cr(VI)), arsenic (As) and mercury (Hg) [35,36]. Additionally, technological equipment such as solar panels and electric-car batteries, which contain high levels of chemical pollutants, have a limited lifespan (10–15 years). If not properly managed, this toxic waste will leach into soil and water, leading to high contamination levels, affecting entire ecosystems and becoming a serious public health threat. Although legislation exists on the disposal of electronic devices, it is known that in many countries these devices still end up in landfills [37,38,39]. On one hand, these materials are problematic as they contain high amounts of toxic elements; on the other, these are the same critical elements necessary to produce new, greener energetic technologies. Although recycling is the most obvious strategy to mitigate this problem, only 17.4% (9.3 Mt) of global electronic waste is recycled [40]. Additionally, several recycling strategies depend on the use of chemical pollutants that produce secondary waste and toxic gases; they can also produce low recovery yields, with associated high costs [40]. Thus, it is imperative to develop more efficient and sustainable recycling strategies that simultaneously mitigate the negative environmental impacts of mining exploration and pollution derived from end-of-life electronic equipment disposal.

### Seaweed-Based Strategies for Heavy Metal Bioremediation and Recycling

With growing awareness of electronic waste as a rising global problem, a significant number of studies on alternative biotechnological approaches, based on natural resources, for recycling and bioremediation purposes can be found in literature [41]. They are expected to play an important role in the energetic transition, enhancing sustainable development, particularly in eco-innovative strategies for electronic waste recycling. Seaweeds are known for their capacity to capture different compounds from polluted scenarios, including highly toxic metallic compounds such as heavy metals. In recent years, significant efforts have been made to evaluate the most suitable conditions and seaweed species for maximum-yield bioremediation at lower costs. Table 3 gathers data concerning advances made in using seaweeds as bioremediation agents in the last 5 years.

Within all classes of seaweeds, brown ones have been the most widely studied [79,80]. This tendency can be seen the last 5 years, with the most commonly studied species being *Ascophyllum* sp., *Colpomenia* sp., *Cystoseira* sp., *Fucus* sp., *Laminaria* sp., *Pelvetia* sp. and *Sargassum* sp., among others. These species have a great capacity to recover metallic pollutants, such as Cd, Pb, Ni, Ag, Cr, Cu, As and Zn, from different sources with high adsorption capacity. Nevertheless, *Sargassum dentifolium* stood out, by removing 99.68% of total hexavalent chromium from water. In addition, *Sargassum filipendula* showed a high capacity to absorb lead, with a maximum uptake of 367.94 mg/g (approximately 96% of total lead) [66,67]. Concerning *Sargassum* species, these attributes gain particular relevance due to the high availably of *Sargassum* biomass all over the world. *Sargassum* species are recognized as highly invasive, impacting non-native ecosystems and being considered a threat to marine biodiversity [80,81]. Thus, developing bioremediation strategies based on *Sargassum* biomass would simultaneously contribute to mitigating the negative impacts of these species.

Another seaweed that stands out, not only for the number of published studies but also due to its high ability to capture different metallic ions from water, is the green seaweed *Ulva lactuca. Ulva lactuca*—also called sea lettuce—is widely abundant in most shorelines around the world, being frequently found in high amounts in sheltered areas [82]. Studies conducted with the dried biomass of these seaweeds recorded 60% to 99% uptake capacity for Cr, Cu, Hg and Pb [45,49,59]. On the other hand, studies conducted with live *Ulva lactuca* also presented high absorption rates of different metallic pollutants, particularly Hg (98%), Pb (87%) and Cu (86%). Since *Ulva lactuca* showed high tolerance to changes in pH, salinity and pollutant concentration, the use of live seaweed may bring additional advantages, particularly in multi-integrated systems.

Although live and dried seaweed biomass have shown high potential for bioremediation purposes, the scale-up to an industrial level presents additional challenges. Most studies are conducted with specific pollutants, in more or less complex mixtures. In real scenarios, the quantity and diversity of chemical pollutants may be extremely high, leading to competitive interactions within the chemical elements, which can require the implementation of sequential remediation steps [80]. However, another seaweed-based strategy may be more suitable for more realistic scenarios—seaweed biochar.

Seaweed waste can be used to produce carbons or biochars, which can have great natural bioremediation potential or can be “engineered” for maximum bioremediation potential [83]. Seaweed biochars also have the great advantage of being able to be recycled, by desorption of the chemical components, and further reused for adsorption purposes. Porous carbons derived from biomass waste have demonstrated an excellent ability to remove several metallic elements from aqueous solutions [84]. Some examples can be found using seaweed biomass to produce value-added porous carbons for adsorption applications [83]; however, no references were found regarding their use as recovery agents of critical raw materials in the last 5 years.

Seaweed biochars are frequently obtained by pyrolysis of the seaweed biomass, resulting in a highly adsorbent, porous, carbon-rich material. Although having a high adsorbent capacity, these carbons can be physically and chemically modified to enhance their specificity and efficiency towards a particular compound, thus presenting important advantages for recycling purposes [83]. Table 4 gathers the most recent works on seaweed biochars/porous carbons for metallic pollutant remediation.

Due to their unique chemical and physical properties, seaweeds are an extremely interesting resource to produce biochar for bioremediation purposes. Seaweeds contain in their composition different minerals, obtained from seawater, such as magnesium, calcium, sodium and potassium, and have generally low carbon content; this affords biochar with a high cation exchangeable capacity, making it highly effective for the uptake of different pollutants, particularly metallic ions. Additionally, seaweed produces biochar with a wide range of microporosity (ranging from <2 nm to >50 nm), which promotes a high adsorption capacity [83,99,100]. Several studies comparing seaweed and terrestrial plant– derived biochar showed that seaweed produce higher amounts of biochar from the same initial biomass, mainly due to its lower lignin content. Moreover, seaweed biochars have been shown to be much more effective in removing metallic ions from aqueous environments, removing up to sixteen times more Zn(II), twelve times more Cd(II) and ten times more Cu(II) than pinewood biochar [94]. Table 4 shows several works in which the use of seaweed-derived biochar revealed high adsorption capacity. Katiyar et al. (2021) evaluated the Cu(II) adsorption capacity of *Ascophyllum nodosum* biochar and verified a removal efficiency of more than 99% from aqueous media, with 223 mg/g Cu(II) adsorption capacity [85]. A kelp-derived biochar showed the capacity to remove more than 90% Cr(III) from an aqueous solution [92].

Seaweed biochar can be chemically modified to enhance its affinity with the target compounds. Biochar uptakes metallic pollutants from water by different mechanisms, including electrostatic interactions, precipitation, complexation, ion-exchange and sorption [83]. Several works have focused in increasing the surface area of biochars to maximize their efficiency. Li et al. (2020) modified a biochar obtained from *Enteromorpha prolifera* by increasing its specific surface area with H_3_PO_4_ and verified a significant increase in Cd(II) uptake from water, with a maximum uptake of 423 mg/g [87]. Kim et al. (2016) used steam to increase *Ulva compressa* biochar surface area and cation exchange capacity and verified an increased efficiency, reaching maximum sorption levels of 137 mg/g Cu(II) [96]. The sorption characteristics of biochar can be altered by different techniques, including changes in pyrolysis time and temperature. Poo et al. (2018) evaluated the effects of different temperatures on *Saccharina japonica* and *Sargassum fusiforme* biochar and verified that temperatures above 400 °C presented higher removal capacity of Cu, Cd and Zn (>98% for *S. japonica*, and >86% for *S. fusiforme*) [94]. By changing the chemical surface functional groups of the *Ulva lactuca* biochar with KOH, Ibrahim et al. (2016) observed an increase of 22% Pb and 30–35% Cd, Cr and Cu absorption, when compared with the same dried seaweed [75].

Several chemical elements are particularly difficult to remove from polluted waters, such as Se, As and Mo. By converting *Oedogonium* sp. and *Gracilaria* sp. biochar into Fe-biochar by slow pyrolysis with FeCl_3_, Johansson et al. (2016) verified an enhanced capacity to adsorb these compounds, with the highest efficiency shown by *Oedogonium* sp., with 67.4, 80.7 and 36.7 mg/g uptake for Mo, As and Se, respectively [89]. In fact, iron-based sorbents are considered one of the most promising approaches for heavy metal polluted scenarios [89,101,102].

## 4. Energy-Storage Devices in the 21st Century

Electrochemical energy conversion and storage technologies such as batteries, fuel cells and supercapacitors are poised to play a significant role in the goal of achieving a carbon-neutral European society by 2050 [2,3,15].

With the growing share of variable renewable energy in power generation with intermittent availability, the need for electricity storage to provide stable loads to the grid has increased significantly. Each of these technologies is critical for the transition towards clean energy and complement one another in reducing greenhouse gas emissions, not only in electricity production but also in transport, industry, and commercial and residential buildings. Thus, energy-storage devices (ESDs), such as supercapacitors, lithium-ion batteries (LIBs) and lithium-ion hybrid capacitors, are key players in the energetic transition.

Although significant advances were achieved in ESD capacity and performance over the last decade [103,104], these cannot respond to current needs, being significantly costly and limited in terms of accumulation efficiency, with a short lifespan.

Due to its properties, namely metallic and non-metallic properties, natural graphite is used in practically all technological applications, including electric vehicle batteries and fuel-cells. With a continuing growth of the electric vehicle industry, the demand for this material has grown exponentially, with it currently considered as a CRM [15].

Carbon materials, such as graphite, are key materials for EED development. It has high electrochemical conductivity and chemical stability, high specific surface area, and low Li reaction potential versus Li/Li+, which is an advantage for high output cell voltage [105]. However, as far as electrochemical and chemical applications are concerned, nanoporosity and surface functionality are the main parameters requiring perfect control. Until now, most of the alternatives available on the market do not meet these requirements. Thus, the search for new materials as sustainable alternatives to graphene has been a rising strategy for high-performance electrochemical energy-storage devices, such as supercapacitors [106].

Biomass-derived porous carbons have particular characteristics that attracted scientists’ interest in testing their use in electronic devices. These materials feature an interconnected nanostructure network, are chemically stable, and have a high specific surface area with light weight, high pore distribution, and high conductivity, which makes them excellent candidates for the development of enhanced electronic devices [107].

### Seaweed—A New Source of Carbons for Electrochemical Applications

Over the last decade, seaweeds have emerged as a low-cost, abundant and sustainable materials for porous carbon production. Due to their chemical composition, such as high protein and polysaccharide content, seaweeds are excellent precursors of nanotextured carbons as electrode materials for electrochemical applications (Table 5).

Among all the studied seaweed, *Enteromorpha* species have shown to be excellent carbon precursors for electrochemical applications. This green seaweed is widely abundant in all coasts around the world and is suitable to be cultured in aquaculture, thus presenting additional advantages with regard to supply and sustainability. Ren et al. (2018) showed that N-doped carbon from *Enteromopha prolifera* has high performance, exhibiting 202 F/g capacitance at 0.5 A/g (700 °C), and a capacitance retention of 96% after 10,000 cycles (800 °C, at 10 A/g current density) [114]. The symmetric supercapacitor displayed a 3000 W/kg power density and 7 Wh/kg energy density, attesting to the potential of *E. prolifera* derived carbons for highly efficient storage devices. Several authors have used brown seaweed biomass to produce carbons. Among them, kelps (Laminariales), comprising more than 30 different species, have a particularly high rate, with some species reaching 30 to 80 m, forming underwater “kelp forests” [135]. Thus, using this biomass as a resource can be a sustainable approach to obtain valuable raw materials for electronic devices. Sun et al. (2019) produced a kelp-derived carbon with a high surface area (2613.7 m^2^/g), which resulted in an enhanced capacitor with high specific capacitance, wide functional voltage range (0.2–7 V), high salt uptake capacity (27.2 mg/g), and rapid response [117]. Zhang et al. (2018) produced a 3D hierarchical porous N, O-doped carbon by the direct carbonization of kelp with a “self-activation” process, and verified an excellent capacitance (669 mF cm^−2^ at 1 mA cm^−2^), with improved ionic storage and transportation in a solid-state symmetric device. This last one demonstrated an excellent capacitance of 412 mF cm^−2^ at 2 mA cm^−2^, and good stability, with 85% retention after 10,000 cycles. The capacitor exhibited a real energy density of more than 0.146 mWh cm^−2^ at 0.8 mW cm^−2^ power density [118]. The process adopted by these authors revealed additional advantages by skipping the activation process and thus reducing the production costs. Cheng et al. (2020) obtained a porous carbon from *Laminaria japonica* with a simple carbonization and activation method and verified that the carbon structure was amorphous, with high heterogenous porosity, high content of oxygen functional groups, and high specific surface area (1902.42 m^2^ g^−1^). This new carbon displayed a specific capacitance of 120 F g^−1^ at 0.1 A g^−1^, with 100% capacitance retention rate after 10,000 cycles [120]. Globally, *Laminaria japonica* carbons showed excellent properties for supercapacitor efficiency improvement.

Within brown seaweeds, *Sargassum* species are widely distributed globally and exhibit an aggressive invasive behavior in many coastlines. One strategy for mitigation of these species’ impacts is their biomass valorization, creating new products that can respond to current challenges [81]. In agreement, several authors have studied the electrochemical properties of *Sargassum*-derived carbons to understand their application in greener supercapacitors. Guo et al. (2021) used *Sargassum* to produce a unique carbon with a micropore spider-web-like structure, by activation with KOH with subsequent melamine nitrogen-doping, under high temperatures. This carbon contained 1.07% N and a high specific surface area (2928.78 m^2^ g^−1^). In a symmetric two-electrode system, the specific capacitance was 481 F g^−1^, with a rate capacity of 85% retention at 10 A g^−1^. In addition, this new N-doped highly porous material promoted an excellent cycling stability, with 100.7% retention rate after 10,000 cycles and a high 16.68 W h kg^−1^ specific energy at 628.9 W kg^−1^, at a current density of 1 A g^−1^ in the voltage range of 0–1 V [127]. Studies have shown that seaweed-derived carbons contain a wide range of porosity (micro, meso and microporous), with a high surface area. These characteristics provide a high ability to uptake and retain sulfur, which can have a practical use for the development of new lithium sulfur batteries (Li-S), which are more efficient than the common lithium-ion batteries due to their higher theoretical energy density, lower cost due to the use of sulfur instead of cobalt [136], and fewer pollutants. Nevertheless, Li-S batteries have the limitations of volume variations with charging cycles, and low sulfur conductivity, which can be mitigated with porous carbon materials [133]. Hencz et al. (2017) developed a highly porous nitrogen-dopped seaweed carbon to enhance the performance of Li-S batteries. The results showed low polarization, good reaction kinetics and excellent rate capabilities, thus showing *Sargassum*-derived carbons to be valuable alternative materials for Li-S improvement [133].

## 5. Final Remarks and Future Perspectives

As a result of humans’ long dependency on fossil-based energies, we are experiencing the impacts of climate change in all ecosystems [137,138]. Thus, it is imperative to pave a way to energetic transition towards carbon-free energy sources. While technological advances are essential for sustainable developed societies, it is also critical to mitigate their associated environmental footprints. Currently, with an exponential demand for greener energetic technologies, there is a great risk of a lack of supply for specific minerals that are essential in all electronic devices and energy storage equipment, which still do not have effective alternatives. On the other hand, the exploration of these valuable minerals results in scenarios of heavy metal soil and water contamination, together with hazardous gas release, resulting from mining activities and chemical purification processes [139].

In the last years, significant efforts have been made in an attempt to respond to these challenges. Within the most recent studies, seaweeds have revealed to be an excellent resource to mitigate some of the negative impacts of energetic transition.

As live sorbents, seaweeds have shown to effectively remove different metallic components from aqueous sources. This approach has been shown to be economic and environmentally sustainable. On the other hand, the use of seaweed biomass can offer additional advantages, such as the reuse of the sorbents in a cycling process, maintaining high absorbent capacity without the need to use costly and toxic chemical solvents in the method.

Due to their particular chemical composition, seaweed biomass affords high yields of carbon materials, which can be directly used for bioremediation purposes as biochars. Although biochars have shown to be highly efficient for metallic pollutant uptake and recycling, these materials can be “engineered” to enhance their affinity towards specific compounds. This approach can bring particular advantages for metal recycling.

Within the scientific advances that are being made in electrochemical devices towards energetic transition, the inclusion of seaweeds in greener energetic technologies is perhaps the most fascinating. Seaweed-derived nanocarbons have shown excellent electrochemical properties as precursors for components of high-performance, carbon-based superconductors, including lithium-ion batteries and fuel cells, as sustainable alternatives to scarce toxic minerals. Additionally, seaweed biomass has shown to have a high biomass conversion efficiency into carbon, with a low impurity content.

Nevertheless, several issues need to be addressed and optimized to reach industrial-scale applications. With regard to bioremediation strategies, since the majority of the studies were conducted under controlled conditions with isolated pollutants, it is important to perform future studies in more realistic scenarios, with real wastewater, to understand the bioremediation efficiency in complex polluted mixtures. For industrial-scale applications of biochars, more research is also needed to decrease the production costs associated with pyrolysis, and simultaneously decrease the need to use toxic chemical compounds in the activation process, which may result in secondary pollution. On the other hand, to guarantee a constant biomass supply, it will be relevant to select cultivable seaweed species or invasive seaweeds. The latter would result in additional environmental advantages, through the mitigation of invasive species negative impacts.

Within a circular economy concept, using the same resource to obtain highly valued materials for different industries will significantly contribute to higher economic and environmental sustainability. Being rich in a panoply of bioactive compounds, minerals and polysaccharides, seaweed derivatives have a wide range of biotechnological applications in chemicals, food, medicinals, feed, agriculture, cosmeceuticals, etc. Seaweed biomass waste derived from bioactive compounds extraction can be valorized into porous carbons and used in the recovery of valuable minerals from liquid effluents. This strategy offers the possibility to reduce both the environmental impacts of mining exploration and processing, as well as of end-of-life electronic equipment disposal, decreasing the reliance on raw mineral extraction. Additionally, seaweed nanocarbons present excellent characteristics to be used as alternative raw materials, for the development of more efficient and cost-effective greener energy-storage devices.

All these characteristics suggest that seaweeds are an important natural resource that can play a key role in energetic transition, to achieve global sustainability in a climate change scenario.

## Figures and Tables

**Table 1:** The 2020 List of Critical Raw Materials in the European Union [8].

Antimony	Coking Coal	LREEs *	PGMs *	Tungsten
Baryte	Fluorspar	Indium	Phosphate rock	Vanadium
Beryllium	Gallium	Magnesium	Phosphorus	Bauxite
Bismuth	Germanium	Natural Graphite	Scandium	Lithium
Borate	Hafnium	Natural Rubber	Silicon metal	Titanium
Cobalt	HREEs *	Niobium	Tantalum	Strontium

* HREEs = Heavy Rare Earth Elements (dysprosium, erbium, europium, gadolinium, holmium, lutetium, terbium, thulium, ytterbium, yttrium). * LREEs = Light Rare Earth Elements (cerium, lanthanum, neodymium, praseodymium, samarium). * PGMs = Platinum Group Metals (iridium, platinum, palladium, rhodium, ruthenium).

**Table 2:** Seaweed-based strategies for the recovery of critical raw materials.

Seaweed	Metal	Maximum Uptake Capacity	Reference
*Cystoseira indica* (xanthated)	La	38.26 mg/g	[16]
*Cystoseira indica* (xanthated)	Ce	41.44 mg/g	[16]
*Fucus spiralis* (live)	Y, La, Ce, Pr, Nd, Eu, Gd, Tb, Dy	ranging from 37% to 61%	[17]
*Fucus vesiculosus* (live)	La, Ce and Eu	>60%	[18]
*Fucus vesiculosus* (live)	Y, La, Ce, Pr, Nd, Eu, Gd, Tb, Dy	55–74%	[17]
*Gracilaria gracilis* (live)	La, Ce, Pr, Gd, and Nd	>60%	[18]
*Gracilaria gracilis* (live)	Y, Ce, Nd, Eu and La.	100%	[19]
*Gracilaria* sp. (live)	Eu	>85%	[20]
*Gracilaria* sp.	Y, La, Ce, Pr, Nd, Eu, Gd, Tb, Dy	ranging from 60 to 93%	[17]
*Gracilaria* sp. (live)	Nd	>90%	[21]
*Hypnea valentiae*	Co	47.44 mg/g	[22]
*Osmundea pinnatifida* (live)	La, Ce	>60%	[18]
*Osmundea pinnatifida* (live)	Y, La, Ce, Pr, Nd, Eu, Gd, Tb, Dy	ranging from 35 to 61%	[17]
*Posidonia oceanica*	Sc	66.81 mg/g	[23]
*Ulva intestinalis* (live)	La, Ce	>60%	[18]
*Ulva intestinalis* (live)	Y, La, Ce, Pr, Nd, Eu, Gd, Tb, Dy	ranging from 63 to 88%	[17]
*Ulva lactuca* (live)	Y, La, Ce, Pr, Nd, Eu, Gd, Tb, Dy	>60%	[18]
*Ulva lactuca* (live)	Eu	>85%	[20]
*Ulva lactuca* (live)	Y, La, Ce, Pr, Nd, Eu, Gd, Tb, Dy	ranging from 80 to 98%	[17]
*Ulva* sp. (live)	Nd	>90%	[21]

**Table 3:** Seaweed-based strategies for recovery of metallic pollutants.

Seaweed	Metal	Maximum Uptake Capacity	Reference
*Ascophyllum nodosum*	Zn(II)	2.34 mmol/g	[42]
*Ascophyllum nodosum*	Cu(II)	20.00 mg/L	[43]
*Caulerpa fastigiata*	Cd(II)	16.48 mg/g (92.01%)	[44]
*Caulerpa racemosa*	Cr	20%	[45]
*Caulerpa racemosa*	Cu	43%	[45]
*Chaetomorpha* sp., *Polysiphonia* sp., *Ulva* sp. and *Cystoseira* sp. (combined)	Zn(II)	115.20 mg/g	[46]
*Codium vermilara*	Cu(II)	>85%	[47]
*Colpomenia sinuosa*	Ni(II) Pb(II) Cd(II)	89%	[48]
*Cystoseira indica*	Cu(II)	30.86 mg/g	[49]
*Cystoseira indica*	U(VI)	250.00 mg/L	[50]
*Cystoseira indica*	Fe(II)	900.00 mg/L	[50]
*Cystoseira indica*	Th(VI)	90.00 mg/L	[51]
*Enteromorpha prolifera*	Cr(VI)	95.25 mg/g	[52]
*Enteromorpha* sp.	Cr(VI)	5.35 mg/g	[53]
*Enteromorpha* sp.	Hg	5.357 mg/g	[54]
*Eucheuma denticulatum*	Pb(II)	81.87 mg/g	[55]
*Eucheuma denticulatum*	Cu(II)	66.23 mg/g	[55]
*Eucheuma denticulatum*	Fe(II)	51.02 mg/g	[55]
*Eucheuma denticulatum*	Zn(II)	43.48 mg/g	[55]
*Fucus spiralis*	Zn(II)	2.04 mmol/g	[42]
*Fucus spiralis* (waste)	Pb(II)	132.00 mg/g	[56]
*Fucus vesiculosus*	Zn(II)	400.00 mg/L	[57]
*Fucus vesiculosus*	Cd	22–76%	[58]
*Fucus vesiculosus*	Pb	65%	[58]
*Fucus vesiculosus*	Pb	86%	[59]
*Fucus vesiculosus* (live seaweed)	Hg	95%	[58]
*Gracilaria changii*	Fe(II)	45%	[60]
*Gracilaria changii*	Cr(VI)	35%	[60]
*Gracilaria changii*	Ni(II)	30%	[60]
*Gracilaria* spp.	Cu(II)	42%	[61]
*Halimeda tuna*	Cu(II)	17.92 mg/g	[49]
*Lyengaria stellata*	Cu(II)	46.29 mg/g	[49]
*Jania rubens*	Ni(II) Pb(II) Cd(II)	91%	[48]
*Laminaria hyperborea*	Zn(II)	2.22 mmol/g	[42]
*Laminaria hyperborea*	Cu(II)	2.50 mg/L	[62]
*Laminaria hyperborea*	Zn(II)	4.30 mg/L	[62]
*Laminaria hyperborea*	Ni(II)	4.20 mg/L	[62]
*Laminaria hyperborea*	Zn(II)	21.5 mg/L	[63]
*Lobophora variegata*	Cu(II)	38.02 mg/g	[49]
*Pelvetia caniculata*	Cr(VI)	2.10 mmol/g	[64]
*Pelvetia caniculata*	Zn(II)	1373.00 mg/L	[64]
*Pelvetia caniculata*	Fe(II)	44.70 mg/L	[64]
*Pelvetia caniculata*	Zn(II)	2.22 mmol/g	[42]
*Sargassum cinereum*	Cu(II)	34.01 mg/g	[49]
*Sargassum dentifolium*	Cr(VI)	~100%	[65]
*Sargassum filipendula*	Cd(II)	103.50 mg/g	[66]
*Sargassum filipendula*	Ni(II)	34.30 mg/g	[66]
*Sargassum filipendula*	Pb(II)	96%	[67]
*Sargassum filipendula*	Ag(I)	0.39 mmol/g	[68]
*Sargassum filipendula*	Cu(II)	0.64 mmol/g	[68]
*Sargassum filipendula*	Pb(II)	367.94 mg/g	[66]
*Sargassum filipendula*	Pb(II)	285.00 mg/g	[67]
*Sargassum glaucescens*	As(III)	207.30 mg/g	[69]
*Sargassum glaucescens*	As(III)	116.60 mg/g	[69]
*Sargassum glaucescens*	As(V)	207.30 mg/g	[69]
*Sargassum glaucescens*	As(V)	116.00 mg/g	[69]
*Sargassum polycystum*	Cd(II)	105.26 mg/g	[70]
*Sargassum polycystum*	Zn(II)	116.20 mg/g	[70]
*Sargassum* sp.	Cd(II)	2.89 mg/g (95%)	[71]
*Sargassum* sp.	Zn(II)	1.85 mg/g (90%)	[71]
*Sargassum* sp.	Cu(II)	95%	[61]
*Sargassum tenerrimum*	Cu(II)	39.84 mg/g	[49]
*Sargassum vulgare*	Fe(III)	20.82 mg/g	[72]
*Ulva compressa*	Cd(II)	95%	[73]
*Ulva fasciata*	Cd(II)	~100%	[74]
*Ulva lactuca*	Cu(II)	60.97 mg/g	[49]
*Ulva lactuca*	Cr	62%	[45]
*Ulva lactuca*	Cu	70%	[45]
*Ulva lactuca*	Hg	96–99%	[59]
*Ulva lactuca*	Pb	86%	[59]
*Ulva lactuca*	Cd	<20%	[59]
*Ulva lactuca*	Ni(II) Cd(II) Pb(II)	85%	[48]
*Ulva lactuca*	Cd(II)	62.5 mg/g	[75]
*Ulva lactuca*	Pb(II)	68.9 mg/g	[75]
*Ulva lactuca*	Cr(III)	60.9 mg/g	[75]
*Ulva lactuca*	Cu(II)	64.5 mg/g	[75]
*Ulva lactuca* (live seaweed)	Hg	98%	[76]
*Ulva lactuca* (live seaweed)	Pb	87%	[76]
*Ulva lactuca* (live seaweed)	Cu	86%	[76]
*Ulva lactuca* (live seaweed)	Ni	77%	[76]
*Ulva lactuca* (live seaweed)	Mn	74%	[76]
*Ulva lactuca* (live seaweed)	Cr	72%	[76]
*Ulva lactuca* (live seaweed)	Cd	56%	[76]
*Ulva lactuca* (live seaweed)	As	48%	[76]
*Ulva ohnoi*	Cd	81%	[77]
*Ulva* sp.	Zn	29.63 mg/g	[78]
*Ulva* spp.	Cu(II)	65%	[61]

**Table 4:** Seaweed-derived biochar/carbons for metallic pollutant uptake.

Seaweed	Metal	Maximum Uptake Capacity	Reference
*Ascophyllum nodosum* biochar	Cu(II)	223.00 mg/g (>99% removal)	[85]
*Enteromorpha prolifera* (magnetically modified biochar)	Cr(VI)	88.17 mg/g	[86]
*Enteromorpha prolifera* (H_3_PO_4_ modified biochar)	Cd(II)	423.00 mg/g	[87]
*Enteromorpha* sp biochar	Cu(II)	91%	[88]
*Enteromorpha* sp biochar	Pb(II)	54%	[88]
*Gracilaria* sp. waste (Fe biochar)	As	62.50 mg/g	[89]
*Gracilaria* sp. waste (Fe biochar)	Mo	78.50 mg/g	[89]
*Gracilaria* sp. waste (Fe biochar)	Se	14.90 mg/g	[89]
*Hizikia* sp. (engineered biochar)	Cd(II)	19.40 mg/g	[90]
*Hizikia* sp. (engineered biochar)	Cu(II)	47.75 mg/g	[90]
*Hizikia* sp. (engineered biochar)	Zn(II)	19.13 mg/g	[90]
*Hizikia fusiformis* biochar	Ni(II)	12.10 mg/g	[91]
*Hizikia fusiformis* biochar	Zn(II)	22.20 mg/g	[91]
*Hizikia fusiformis* biochar	Cu(II)	2.24 mg/g	[91]
*Hizikia fusiformis* biochar	Ld(II)	2.89 mg/g	[91]
*Hizikia fusiformis* biochar	Cd(II)	22.00 mg/g	[91]
Kelp (engineered biochar)	Cd(II)	23.16 mg/g	[90]
Kelp (engineered biochar)	Cu(II)	55.86 mg/g	[90]
Kelp (engineered biochar)	Zn(II)	22.22 mg/g	[90]
Kelp biochar	Cr(III)	39.16 mg/g (91.13%)	[92]
*Oedogonium* sp. (Fe biochar)	Mo	67.40 mg/g	[89]
*Oedogonium* sp. (Fe biochar)	As	80.70 mg/g	[89]
*Oedogonium* sp. (Fe biochar)	Se	36.80 mg/g	[89]
*Porphyra tenera* biochar	Cu(II)	75.10 mg/g	[93]
*Porphyra tenera* biochar (steam activated)	Cu(II)	~78.00 mg/g	[93]
*Porphyra tenera* biochar (KOH-activated)	Cu(II)	75.10 mg/g	[93]
*Saccharina japonica* biochar	Cu(II	98.60 mg/g (>98%)	[94]
*Saccharina japonica* biochar	Cd(II)	60.70 mg/g (>98%)	[94]
*Saccharina japonica* biochar	Zn(II)	84.30 mg/g (>98%)	[94]
*Sargassum fusiforme* biochar	Cu(II	94.10 mg/g (>86%)	[94]
*Sargassum fusiforme* biochar	Cd(II)	37.20 mg/g (>86%)	[94]
*Sargassum fusiforme* biochar	Zn(II)	43.00 mg/g (>86%)	[94]
*Sargassum* sp.	Hg	7.41 mg/g	[54]
*Turbinaria turbinata* biochar	Cr(VI)	12.60 mg/g	[95]
*Ulva compressa* biochar (steam activated)	Cu(II)	137.00 mg/g	[96]
*Ulva lactuca* KOH activated carbon	Cu (II)	84.70 mg/g	[75]
*Ulva lactuca* KOH activated carbon	Cr(III)	81.90 mg/g	[75]
*Ulva lactuca* KOH activated carbon	Cd(II)	84.60 mg/g	[75]
*Ulva lactuca* KOH activated carbon	Pb(II)	83.30 mg/g	[75]
*Ulva lactuca* biochar	Pb(II)	3.49 mg/g	[97]
*Ulva reticulata* biochar	Ar (V)	8.12 mg/g	[98]

**Table 5:** Seaweed-derived carbons for high-performance energy-storage device development.

Seaweed	Preparation of Seaweed Biochar	Main Achievements	Reference
*Ascophyllum nodosum*	- Carbonization: 700 °C in alumina crucible, 2 h, under N_2_ flow - Activation: KOH and HCl	- High surface area of 1493 m^2^/g and a current density of 5.2 mA/cm^2^ - Capacitance of 207.3 F/g at 0.5 A/g and a good stability after 2500 cycles at 5 A/g with a retention capacity of 92.3%	[108]
*Cladophora glomerata*	- Carbonization: 900 °C, under Ar flow - Activation: HNO_3_	- High specific capacitances of raw and treated samples of 201 F/g and 392 F/g at 5 mV/s, respectively - Energy densities of 22.2 Wh/kg and 42.4 Wh/kg at 450 w/kg, respectively - High retention of 101.9%, after 5000 cycles - The activation with HNO_3_ showed an enhancing influence on the supercapacitive of the electrodes	[109]
*Enteromorpha clathrate*	- Carbonization: 800 °C, 1 h - Activation: KOH and ZnCl_2_	- ZnCl_2_ is an efficient activation agent for seaweed to make hierarchical structures when compared to KOH - Excellent gravimetric capacitance of 207.6 F/g at 1 A/g	[110]
*Enteromorpha prolifera*	- Carbonization: 850 °C, 3 h under N_2_ flow	- Capacitance of 180 F/g, and the specific capacitance retention was 96% after 2000 cycles - Excellent electrochemical performance	[111]
*Enteromorpha prolifera*	- Carbonization: 500 °C, 2 h, under air in a tube furnace - Activation: KOH	- High specific surface area of 3536.58 m^2^/g - High sulfur loading (74.8%) - The specific capacity was 530 mAh/g after 100 cycles - Candidate for use as the cathode material in lithium-sulfur batteries	[112]
*Enteromorpha prolifera*	- Carbonization: 500 °C, 2 h under N_2_ flow - Activation: KOH, at 600–800 °C, 1 h, under N_2_ flow	- Highest surface area of 3345 m^2^/g and highest pore volume of 1.94 cm^3^/g - Highest capacitance (800 °C) of 440 F/g at 1 A/g in 6M KOH and retention of 87% after 5000 cycles	[113]
*Enteromorpha prolifera*	- Carbonization: 450 °C, 2 h, under N_2_ flow - Activation: KOH, at 600–800 °C, 1 h	- Surface area of up to ~2000 m^2^/g with N-species content of ~2.9 at % and pores of less than 3.0 nm - High performance, displaying 202 F/g capacitance at 0.5 A/g (700 °C) - Capacitance retention of 96% after 10,000 cycles (800 °C), at 10 A/g current density, in 6M KOH - N-doped carbon shows promising perspective for supercapacitor technology	[114]
*Enteromorpha prolifera*	- Carbonization: 600 °C, 3 h, under N_2_ flow - Activation: ZnCl_2_ with different ratios	- The samples obtained at ratio 4 exhibit the highest specific surface area of 1910.84 m^2^/g and largest total pore volume of 2.68 cm^3^/g - High specific capacitance of 167 F/g in 6M KOH and 332.4 F/g in 1M H_2_SO_4_ - High capacitance retention of 90.32% and 73.9%, after 20,000 cycles in 6M KOH and 332.4 F/g in 1M H_2_SO_4_, respectively - Retention of 100% after 5500 cycles - Excellent candidate for energy storage	[115]
*Enteromorpha* sp.	- Carbonization: 800 °C, 1 h, under N_2_ flow - Activation: KOH	- High specific capacitance of 201 F g^−1^ (10.7 µF cm^−2^) at 1 A g^−1^ and 20 °C - Capacitance retention ratio of 61% at 100 A g^−1^ - Capacitance loss of 9% after 10,000 cycles	[116]
Kelp	- Carbonization: 700 °C, 1 h - Activation: KOH	- Material with a high specific surface area (2613.7 m^2^ g^−1^), hierarchical structure, and excellent conductivity - Outstanding capacitance storage feature (202F) at a current density of 1.0 A and long-time stability	[117]
Kelp	- Carbonization: 600–900 °C, 3 h, under N_2_ flow - Activation: “self-activated”	- 3D hierarchical porous N, O-doped carbon delivered excellent capacitance of 669 mF cm^−2^ at 1 mA cm^−2^ - A flexible solid-state symmetric device showed: - Capacitance of 412 mF cm^−2^ at 2 mA cm^−2^ - Cyclic stability with the retention of 85% after 10,000 cycles	[118]
Kelp	- Carbonization: 900–1600 °C, 2 h, under Ar flow	- High capability (a stable capacity of 96 mAh g^−1^ at 1000 mA g^−1^) - Excellent cycling performance (205 mAh g^−1^ after 300 cycle at 200 mA g^−1^) - Good specific capacity at potentials higher than 0.05 V	[119]
*Laminaria japonica*	- Carbonization: 600–1200 °C, 2 h, under N_2_ flow - Activation: KOH	- Successful use as an electrode for supercapacitors - Highest specific surface area (2088.31 m^2^/g) and total pore volume (1.38 cm^3^/g) at 800 °C - At 700 °C, the capacitance retention rate and coulomb efficiency are close to 100%, even after 10,000 cycles at 1 A/g	[120]
*Laminaria japonica*	- Carbonization: 500 °C, 1 h, under N_2_ flow - Activation: KOH, at 900 °C, 1 h, under N_2_ flow	- High capacitance of 381 and 268 F/g (1 and 50 A/g) in 6 mol/L KOH and 382 and 160 F/g (1 and 50 A/g) in 1 mol/L H_2_SO_4_ - Good rate capacity, great specific capacitance, and long-term cycling stability	[121]
*Lessonia trabeculata*	- Carbonization: 800 °C, 1 h, under N_2_ flow - Activation: KOH	- The activated process leads to enhancement of specific surface area of 769 m^2^/g - Good capacity retention of 96% after 500 cycles	[122]
Nori	- Pretreatment: ZnCl_2_ - Carbonization: 700–800 °C, 2 h, under N_2_ flow - Activation: KOH	- High capacitive performance of 220 F/g, good rate capability of 61.5% from 0.1 to 10 A/g - Very high specific volumetric capacitance of 307.7 F/cm^3^ - High-performance supercapacitors	[123]
*Porphyra sp.*	- Activation: Ni(NO_3_)_2_ - Carbonization: 700–1000 °C, 2 h, under Ar flow	- Stable and reversible capacity of 352 mAhg^−1^ at 10 Ag^−1^ with retention of 43% - Capacity of 348 mAhg^−1^ after 3000 cycles at 5 Ag^−1^ with retention of 81%	[124]
*Sargassum muticum*	- Pretreatment: H_3_PO_4_ - Carbonization: 600 °C, 2 h, under N_2_ flow - Activation: KOH, at 350 °C, 30 min	- Excellent pore structures and high graphitization - High specific capacitance of 511 F/g - Good stability, capacity retention of 90% after 5000 cycles	[125]
*Sargassum* sp.	- Pretreatment: NH_3_·H_2_O - Carbonization: 600 °C, 3 h, under N_2_ flow - Activation: KOH	- Highest surface area of 3251.42 m^2^/g - High gravimetric specific capacitance of 336 F/g and a good rate capacity of 82% retention at 10 A/g - High cycling capacity of 85% after 10,000 cycles at a current density of 5 A/g	[126]
*Sargassum* sp.	- Carbonization: 600 °C, 3 h, under N_2_ flow - Activation: KOH and addiction of melamine (nitrogen-doping)	- The structure and electrochemical performance are influenced be the N-doping amount - High specific surface area of 2928.78 m^2^/g (nitrogen content of 1.07%) - High gravimetric specific capacitance of 481 F/g in 6M KOH and a good rate capacity of 85% retention at 10 A/g - Good capacitance retention of 100.7%, after 10,000 cycles	[127]
*Sargassum* spp.	- Carbonization: 700 °C, 90 min, under N_2_ flow - Activation: KOH	- High current density at 0.2 V and an onset potential of 0.852 V - BET surface area of 133.871 m^2^ g^−1^	[128]
*Sargassum wightii*	- Carbonization: 700–900 °C in alumina crucible, 3 h, under Ar flow	- The sample (700 °C) reveals the maximum capacitance of 354 F/g at 0.5 A/g in 1M H_2_SO_4_ - This clearly reflects that seaweed can be used as supercapacitors	[129]
*Turbinaria cunoides*	- Carbonization: 700–900 °C, in alumina crucible, 3 h, under Ar flow	- Low surface area 173.8 m^2^/g. High specific capacitance of 416 F/g at the current density of 1 A/g - High energy capacity 52 Wh/kg at a powder density of 104 W/kg - 85.3% of capacitance after 5000 cycles	[130]
*Ulva fasciata*	- Carbonization: 700–900 °C, 3 h, under N_2_ flow	- High electrical conductivities of 9100 mS/m and surface area of 376 m^2^/g - High gravimetric capacitance (800 °C) of 330 F/g with a powder density of 10 kW/kg - Capacitance retention of 97.5% after 5000 cycles	[131]
*Ulva lactuca*	- Carbonization: 850 °C, 4 h, under N_2_ flow - Activation: HNO_3_	- Used for supercapacitor - Superior electrochemical performance	[132]
Brown seaweed	- Carbonization: 800 °C, 2 h, under Ar flow - Activation: HCl	- Prepared seaweed carbon was employed in Li-S batteries—High initial discharge capacity of 1200 mAh g^−1^ at 0.2 °C and a good reversible capacity of 575 mAh g^−1^ at 1 °C, over 300 cycles - Beneficial chemical, physical morphology, and excellent electrochemical performances	[133]
Seaweed Biomass	- Carbonization: 450 °C, 4 h, under N_2_ flow - Activation: KOH	- Improved electrochemical performance with good specific capacity and retention, long-term cyclability, and rate capability - Energy density of 163 Wh kg^−1^	[134]

## Data Availability

Not applicable.
